# Health literacy and health outcomes among older patients suffering from chronic diseases: A moderated mediation model

**DOI:** 10.3389/fpubh.2022.1069174

**Published:** 2023-01-10

**Authors:** Jinjin Lu, Shuting Sun, Yechun Gu, Huihui Li, Liangyu Fang, Xiaoling Zhu, Hongbo Xu

**Affiliations:** ^1^School of Nursing, Wenzhou Medical University, Wenzhou, Zhejiang, China; ^2^General Surgery Department, Wenzhou Hospital of Integrated Traditional Chinese and Western Medicine, Wenzhou, Zhejiang, China; ^3^School of Public Health and Management, Wenzhou Medical University, Wenzhou, Zhejiang, China

**Keywords:** health literacy (MeSH), self-efficacy, health outcomes, chronic disease, aged

## Abstract

**Introduction:**

Aging brings with an increased risk of chronic diseases among older adults, which could affect health outcomes. Evidence has showed that health literacy is associated with health outcomes. However, limited studies explore the underlying mechanism between health literacy and health outcomes. Hence, this study aimed to determine whether self-efficacy for managing chronic disease mediates the relationship between health literacy and health outcomes among older patients with chronic diseases, and to explore whether disease duration moderates the relationship between health literacy, self-efficacy for managing chronic disease, and health outcomes.

**Methods:**

Participants were recruited from tertiary hospitals in Zhejiang Province, China from May 2019 to June 2020 using a convenience sampling method. A total of 471 older patients with chronic diseases completed questionnaires measuring demographics, disease-related information, health literacy, self-efficacy for managing chronic disease, and health outcomes. The mediation effect was examined using the structural equation model method, based on the bias-corrected bootstrapping method. The moderation effect was tested by the multiple-group analysis.

**Results:**

A good fit model suggested that self-efficacy for managing chronic disease partially mediated the relationships between health literacy and health outcomes. In addition, disease duration moderated the relationships between health literacy, self-efficacy for managing chronic disease, and health outcomes.

**Discussion:**

The findings highlight that adequate health literacy improved health outcomes among older patients with chronic diseases, which was further promoted by self-efficacy for managing chronic diseases. Moreover, a long disease duration could enhance the effect.

## Introduction

Chronic disease is increasingly recognized as a serious, worldwide public health concern. Every year, 41 million people die from chronic diseases, which is equivalent to 71% of all deaths globally (1). In China, the prevalence of chronic diseases is increasing. A report from a nationwide survey showed that more than 20% of people aged 18 years and above suffer from at least one chronic disease (2). More concerning, chronic diseases accounted for four-fifth of total deaths, and continue to become the predominant disease burden (3). With the aging population and the globalization of unhealthy lifestyles, older people are particularly at risk of chronic diseases. Chronic diseases tend to be of long duration, imposing physical and mental burdens on the elderly and affecting their health outcomes.

Health literacy is defined as the extent to which individuals possess the knowledge, motivation, and competencies to access, understand, appraise, and apply health information (4). Researchers have found that adequate health literacy generally contributes to improving health outcomes. For example, a meta-analysis showed that health literacy is significantly associated with better diabetes outcomes, such as glycemic control, knowledge, and self-care (5). Severaleveral studies indicated that adequate health literacy is more likely associated with greater health-related quality of life and self-rated health (6). Conversely, limited health literacy is strongly associated with worse health outcomes, which leads to a higher risk of mortality, hospitalizations, and healthcare cost (7–11). Solid evidence has confirmed that there is a significantly positive association between health literacy and health outcomes, but how and when it affects health outcomes in older adults with chronic diseases needs to be further explored. Answering these questions will help healthcare professionals conduct targeted and effective interventions to promote older patients' health outcomes.

According to Bandura's self-efficacy theory, self-efficacy is one's belief in the ability to organize and execute actions required to produce given levels of attainments (12). A high sense of self-efficacy will bring stronger intentions to complete a behavior. Specifically, patients with strong self-efficacy tend to complete health-related behaviors and ultimately get better health outcomes. In a conceptual framework for explaining health outcomes, Ussher proposed the association between health literacy, self-efficacy, and health outcomes. They predicted that individuals with higher health literacy would have stronger self-efficacy (13). A number of studies also have found a positive association between health literacy and self-efficacy (14–17). In addition, the association between self-efficacy and health outcomes has also been identified. Previous research showed that higher self-efficacy is significantly associated with better blood pressure control, glycemic control, and medication adherence (18, 19). Evidence has found support for the potential mediating role of self-efficacy on health outcomes. A study found that self-efficacy played a partial mediating effect on the relationship between medication literacy and medication adherence (20). Accordingly, we speculated that health literacy could enhance health outcomes by improving the patients' self-efficacy for managing chronic disease. Therefore, we proposed the first hypothesis: self-efficacy for managing chronic disease plays a mediating role between health literacy and health outcomes.

High health literacy may result in good health outcomes through the mediating role of self-efficacy, but not all individuals with high health literacy homogeneously experience high levels of self-efficacy and have good health outcomes. Though lack of direct evidence, indirect evidence suggested that disease duration may moderate the relationship between health literacy and health outcomes. A study found that diabetes duration was associated with diabetic health literacy (21). Patients with inadequate health literacy were attained with shorter diabetes duration. Inversely, research has shown that long disease duration was negatively related to good glycemic control among patients with type 2 diabetes mellitus (22, 23). Therefore, we proposed the second hypothesis: disease duration plays a moderating role between health literacy and health outcomes.

Disease duration may moderate not only the direct relationship between health literacy and health outcomes but also the mediating effect of self-efficacy. According to the previous evidence, disease duration was an important influencing factor for both health literacy and self-efficacy. A study on patients with hypertension showed that patients with long duration of hypertension have better medication literacy comparing those with short duration (20). Studies also found that disease duration was positively associated with self-efficacy among patients with type 2 diabetes mellitus. Patients with long disease duration had stronger self-efficacy than those with shorter duration (24, 25). In this study, we speculated that the positive association between health literacy and self-efficacy was stronger in individuals with longer disease duration and weaker in individuals with shorter disease duration. Therefore, we proposed the third hypothesis: disease duration plays a moderating role between health literacy and self-efficacy in managing chronic disease. Moreover, disease duration may also moderate the relationship between self-efficacy and health outcomes. A meta-analysis found that pain duration had a significant moderating effect on the association between self-efficacy and chronic pain outcomes (26). Additionally, the moderating effect of pain duration had a larger effect on the patients having a longer duration than those having a shorter duration. Thus, we speculated that patients with long disease duration could strengthen the positive effect of self-efficacy on health outcomes. Therefore, we proposed the fourth hypothesis: disease duration plays a moderating role between self-efficacy for managing chronic disease and health outcomes.

**Figure 1:**
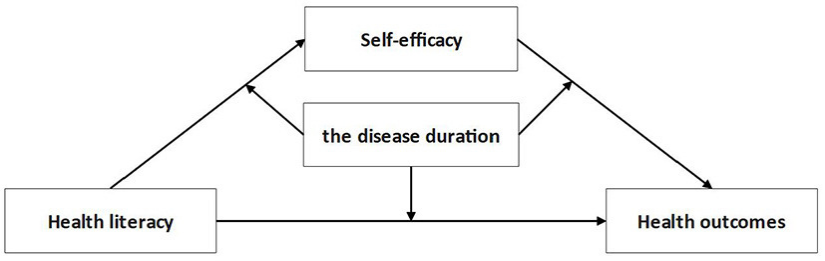
The proposed moderated mediation model.

In summary, this study aimed to further explore the relationship between health literacy and health outcomes among older patients with chronic diseases. Specifically, it included the following: first, whether self-efficacy for managing chronic disease plays a mediating role between health literacy and health outcomes; second, whether disease duration moderates the first and second half and direct path of the mediating relationship. Our hypothesized model can be seen in Figure 1.

## Materials and methods

### Study design and participants

A cross-sectional study was conducted in tertiary hospitals in Zhejiang Province, China, which was from May 2019 to June 2020. In this study, a total of 520 patients were recruited using the convenience sampling method. The inclusion criteria for the participants were as follows: (a) aged 60 years and older; (b) have confirmed at least one diagnosis of chronic diseases. The exclusion criteria for the participants were patients who had been diagnosed with: (a) mental disorders (e.g., Schizophrenia, Alzheimer's disease, Bipolar disorder); (b) malignancy. Before data collection, the investigators would explain the purpose of the study to patients and obtain informed consent from each patient. Then, the investigators used article questionnaires to conduct face-to-face surveys. After removing invalid questionnaires, 471 valid questionnaires were included in the analysis ultimately.

The study sample size was calculated using G^*^Power 3.1.9.7 with 50% effect, 5% precision, and 90% power, giving a sample size of 298. In addition, structural equation modeling was used to test the moderated mediation effect in this study. According to previous studies, structural equation modeling requires the sample size to be 10–15 times the number of variables (27). This study contained a total of 23 variables (10 demographic items, four health literacy dimensions, one self-efficacy variable, and eight health outcomes domains). Considering 10% non-responders, the final sample size was set at 256–383. The actual sample is 471 which size is satisfactory for analysis.

### Measurements

The questionnaire included demographic information, disease-related information, and three scales selected according to the purpose of the study. Demographic information included age, gender, marital status, occupation before retirement, the number of children, living arrangements, education attainment, and monthly household income. Disease-related information was regarding the number of chronic diseases and disease duration. The basic information of the three scales is as follows.

#### Health literacy

Health literacy was measured by a health literacy scale for chronic patients. The scale was developed by Jordan et al. (28) and translated by Sun (29). It consists of 24 items about the patients' information acquisition ability, communication and interaction ability, health improvement willingness, and economic support willingness. Each item is rated on a 5-point scale from 1 (with great difficulty or reluctance) to 5 (without any difficulty or strongly willing). The total score ranges from 24 to 120, with higher scores implying greater health literacy. The scale showed acceptable internal consistency (Cronbach's α = 0.815) that was in line with prior research (Cronbach's α = 0.894) (30).

#### Self-efficacy for managing chronic disease

Self-efficacy for managing chronic disease was assessed by the Chinese version of the Self-efficacy for Managing Chronic Disease scale (SEMCD) (31). The scale includes six items on a 10-point scale from 1 (not at all confident) to 10 (totally confident). The score is computed as the mean of the six items. Higher scores indicate greater self-efficacy. The instrument demonstrated high reliability and validity. The Cronbach's α value of 0.938 obtained in the current study indicated acceptable internal consistency (31).

#### Health outcomes

Health outcomes were measured by the Chinese version of the 12-item Short-Form Health Survey (SF-12). The scale includes the following eight domains: general health, physical functioning, role-physical, bodily pain, vitality, social functioning, role-emotional, and mental health. The physical and mental health of the older persons was investigated through the physical component summary (PCS) and mental component summary (MCS) of the scale, respectively. The scores were calculated by summing items and then transforming these raw scores to a 0–100 scale using norm-based methods with a mean of 50 and a standard deviation of 10. Higher scores indicate better health. The SF-12 PCS and MCS scales demonstrated good internal consistency, with Cronbach's α ≥ 0.82 and 0.75, respectively (32). In this study, Cronbach's α coefficient of the questionnaire was 0.915.

### Ethical considerations

The study was approved by the Ethics Committee of the First Affiliated Hospital of Wenzhou Medical University (KY2021-104). Before the interviews, informed consent was obtained. All participants were informed that this study was anonymous and did not pose any harm to them.

### Data analysis

Data management and analysis were performed using SPSS 26.0 and AMOS 22.0. Continuous variables were described with means and standard deviation. Categorical data were presented as numbers and percentages. Participants were divided into two groups according to their disease duration, which was < 10 years. This grading standard was mainly based on the previous evidence (33). First, descriptive statistics were generated for all variables. Second, univariate analysis was undertaken to compare the differences between the two groups. Continuous data were analyzed with the independent-sample *t*-test or the Kruskal–Wallis test. Categorical data were performed with Chi-square for nominal distribution and the Kruskal–Wallis test for ordinal distribution as appropriate. Then, a structural equation model was carried out to determine the direct effect of health literacy on health outcomes and the role of self-efficacy in managing chronic disease as a mediator in this relationship. Given the statistically significant variables in univariate analysis, variables including age, gender, occupation, and the number of chronic diseases were taken as covariates. A bias-corrected bootstrap confidence interval was employed to test the indirect mediation effects. Next, the multiple-group analysis was used to assess the moderating effect of the disease duration. A *p* < 0.05 was accepted to be statistically significant.

## Results

### Descriptive statistics

In total, 471 participants were enrolled in this study, of whom 231 (49%) were men and 240 (51%) were women. Of the 417 participants, 219 were with disease duration of ≥10 years, and 252 were with disease duration of < 10 years. The demographic characteristics of participants are presented in Table 1. There was no difference between the two groups in the with respect to the marital status, the number of children, living arrangements, education attainment, and monthly household income of the participants. Only four demographic variables including age, gender, occupation before retirement, and the number of chronic diseases were significantly different between the two groups.

**Table 1:** Descriptive statistic of the participants (*N* = 471).

**Classification**		**Total (*N* = 471)**	**Disease duration < 10 years (*N* = 252)**	**Disease duration ≥10 years (*N* = 219)**	**Test statistic**	***P***
		***M (SD) or N (%)***				
Age	60–69	183 (38.9)	111 (44.0)	72 (32.9)	12.753^a^	<0.001
	70–79	195 (41.4)	108 (42.9)	87 (39.7)		
	≥80	93 (19.7)	33 (13.1)	60 (27.4)		
Gender	Male	231 (49.0)	108 (42.9)	123 (56.2)	8.303^b^	0.004
	Female	240 (51.0)	144 (57.1)	96 (43.8)		
Marital status	Married	399 (84.7)	213 (84.5)	186 (84.9)	0.015^b^	0.902
	Divorced or widowed	72 (15.3)	39 (15.5)	33 (15.1)		
Occupation before retirement	Self-employment	60 (12.7)	36 (14.3)	24 (10.9)	9.690^b^	0.046
	Freelance	42 (8.9)	30 (11.9)	12 (5.5)		
	Farmer	216 (45.9)	114 (45.2)	102 (46.6)		
	Staff of government agencies or public sector	84 (17.8)	42 (16.7)	42 (19.2)		
	Worker	69 (14.7)	30 (11.9)	39 (17.8)		
No. of children	0–2	162 (34.4)	93 (36.9)	69 (31.5)	1.513^b^	0.219
	≥3	309 (65.6)	159 (63.1)	150 (68.5)		
Live alone	No	399 (84.7)	210 (83.3)	189 (86.3)	0.797^b^	0.372
	Yes	72 (15.3)	42 (16.7)	30 (13.7)		
Educational attainment	None	165 (35.0)	75 (29.8)	90 (41.1)	2.112^a^	0.146
	Primary school	189 (40.1)	114 (45.3)	75 (34.2)		
	Middle school	42 (8.9)	27 (10.7)	15 (6.9)		
	High school or secondary vocational school	30 (6.4)	18 (7.1)	12 (5.5)		
	Undergraduate or junior college above	45 (9.6)	18 (7.1)	27 (12.3)		
Monthly household income (RMB)	≤1,500	54 (11.5)	24 (9.5)	30 (13.7)	0.238^a^	0.625
	1,501–3,000	168 (35.7)	90 (35.7)	78 (35.6)		
	3,001–5,000	171 (36.3)	102 (40.5)	69 (31.5)		
	5,001–10,000	48 (10.2)	21 (8.3)	27 (12.3)		
	>10,000	30 (6.4)	15 (6.0)	15 (6.9)		
No. of chronic disease	1	198 (42.0)	135 (53.6)	63 (28.8)	29.586^b^	<0.001
	≥2	273 (58.1)	117 (46.4)	156 (71.2)		
Health literacy		81.7 (22.8)	83.6 (20.8)	79.6 (24.7)	1.874^c^	0.062
Self-efficacy		45.2(12.7)	45.7 (11.5)	44.7 (13.9)	0.854^c^	0.394
PCS		52.5 (25.9)	54.3 (23.2)	50.3 (28.7)	1.673^c^	0.095
MCS		64.1 (18.2)	66.5 (17.2)	61.3 (18.9)	3.166^c^	0.002

PCS, physical component summary; MCS, mental component summary;

a Z;

b χ^2^;

c t.

### Mediation analysis

**Figure 2:**
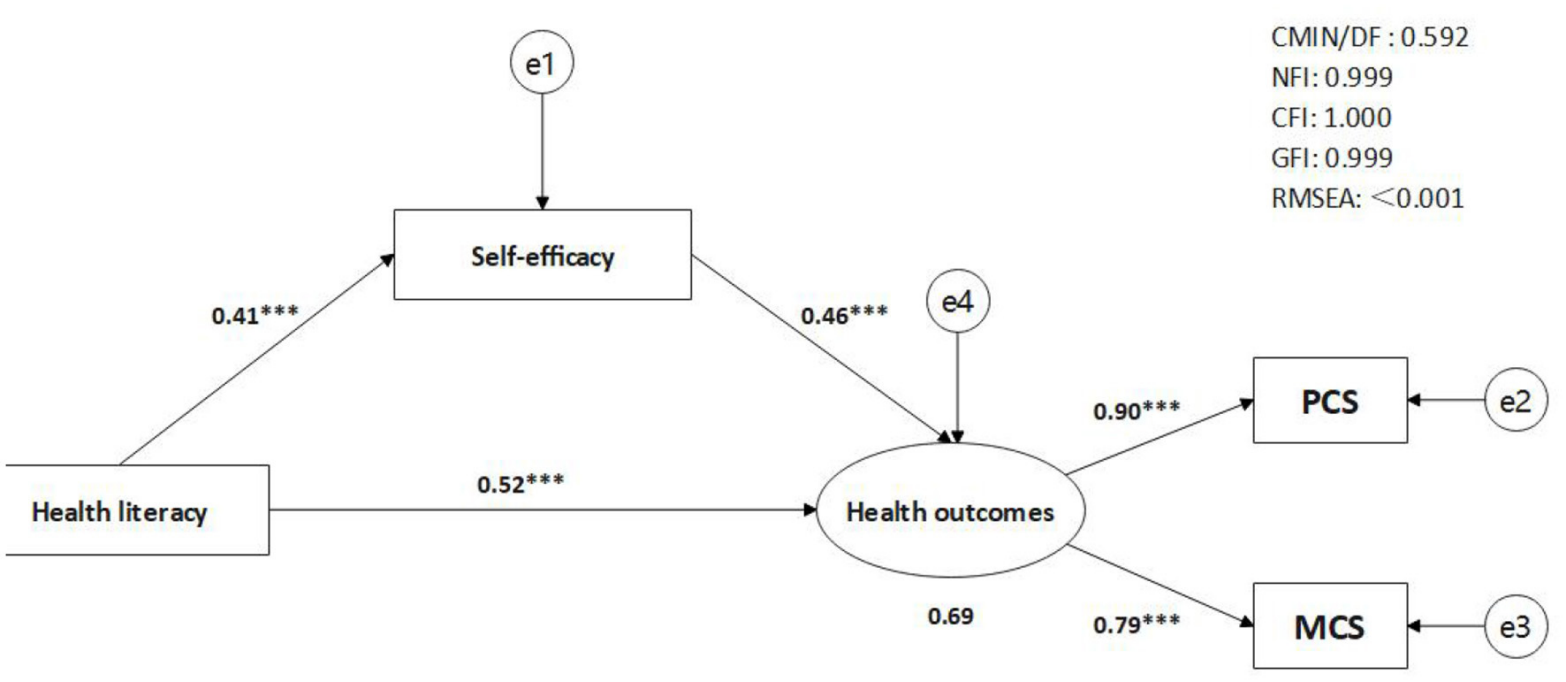
The mediation model. PCS, physical component summary; MCS, mental component summary. ***p < 0.001.

The proposed mediation model revealed a good fit to the data (χ^2^/df = 0.59, CFI = 1.00, NFI = 0.99, GFI = 0.99, RMSEA < 0.001, see Figure 2). Health literacy was significantly and positively associated with self-efficacy for managing chronic disease (β = 0.41, *p* < 0.001), and the latter was also significantly and positively associated with health outcomes (β = 0.46, *p* < 0.001). As expected, the direct effect of health literacy on health outcomes was significant [β = 0.52, 95% CI = (0.446, 0.591), *p* < 0.001]. The results of bootstrapping test revealed that self-efficacy for managing chronic disease partially mediated the relationship between health literacy and health outcomes, and the indirect effect was 0.192 [95% CI = (0.147, 0.242)]. The mediated effect accounted for 26.9% of the total effect (see Table 2).

**Table 2:** The mediating effect of self-efficacy on health literacy and health outcomes.

	**Effect**	**95% BCI**
Direct effect	0.522^***^	0.446–0.591
Indirect effect	0.192^***^	0.147–0.242
Total effect	0.714^***^	0.655–0.808

BCI, Bootstrapped confidence interval;

*** *p* < 0.001.

### Moderated mediation analysis

The results showed that the moderated mediation model fit the data well (see Appendix A). Disease duration moderated the above mediation model (see Table 3, Appendix B). The results revealed that disease duration moderated the direct effect of health literacy on health outcomes. The path from health literacy to health outcomes was significantly stronger among the participants with disease duration ≥10 years [β = 0.551, 95% BCI = (0.473, 0.625)] than those with disease duration < 10 years [β = 0.496, 95% BCI = (0.379, 0.601)] (Δχ^2^/df = 31.919, Δdf = 9, *p* < 0.001). Besides, the results confirmed our hypothesis explaining that disease duration moderated the association between health literacy and self-efficacy for managing chronic disease (β = 0.475, *p* < 0.001 *for* disease duration ≥10 years vs. β = 0.336, *p* < 0.001 for disease duration < 10 years), as well as the path between self-efficacy for managing chronic disease and health outcome (β = 0.490, *p* < 0.001 for disease duration ≥10 years vs. β = 0.401, *p* < 0.001 for disease duration < 10 years). It illustrated the indirect effect of health literacy on health outcomes was more obvious in individuals with a duration of ≥10 years.

**Table 3:** The moderating effect of disease duration.

**Dependent variable**	**Independent variable**	**Disease duration**<**10 years**		**Disease duration** ≥**10 years**	
		β	**95% BCI**	β	**95% BCI**
Self-efficacy	Health literacy	0.336	0.223–0.447	0.475	0.357–0.571
Health outcomes	Self-efficacy	0.401	0.255–0.524	0.490	0.390–0.588
Health outcomes	Health literacy	0.496	0.379–0.601	0.551	0.473–0.625

## Discussion

This study revealed that health literacy was significantly and positively associated with health outcomes among older patients with chronic diseases. This finding was consistent with previous research results (5, 6). The results further indicated that self-efficacy for managing chronic disease played a mediation role between health literacy and health outcomes, which verified the first hypothesis. Moreover, it verified the conceptual framework of mechanisms linking health literacy to health outcomes. The framework considers that individuals with higher health literacy would have stronger self-efficacy and could obtain better health outcomes. Bandura believed that self-efficacy is an important determinant of intention and behaviors (12). When patients are convinced that they were capable of managing their own diseases, they would have a high sense of self-efficacy to participate in disease management activities, which would affect both physical and mental health (18, 19). In other words, older patients who experience strong self-efficacy for managing chronic diseases are more likely to perform health behaviors using their health-related knowledge. Thus, with an increase in health literacy, the older would be more confident in managing their health-related behaviors, which would affect health outcomes including physical and mental. This study confirmed that health literacy could affect health outcomes by strengthening the self-efficacy of older adults for managing chronic diseases. Therefore, healthcare professionals should enhance older adults' self-efficacy for managing chronic diseases and help them equip with adequate health literacy to achieve better physical and mental health through self-managing behaviors.

This study found that disease duration moderated the relationship between health literacy and health outcomes, and the first and second half of the mediating effect of self-efficacy for managing chronic disease. Thus, the results supported the previous hypothesis. As known, chronic diseases tend to be of long duration and people with chronic diseases need long-term care. Previous evidence showed that the disease duration could influence chronic disease management (34). First, in this study, the positive relationship between health literacy and health outcomes was more significant in patients with long disease duration. The results revealed that disease duration played a positive role at this time, which was in line with previous results (23, 35). This could be because patients with long disease duration might increase the probability of health-related stimulus to strengthen the positive relationship between health literacy and health outcomes. Therefore, these results supported the second hypothesis. Second, the results demonstrated that disease duration had a significant moderating effect on the relationship between health literacy and self-efficacy for managing chronic disease. In individuals with long disease duration, the promoting effect on self-efficacy for managing chronic disease was more evident with an increased health literacy. That is, patients with long disease duration might be exposed to more health-related knowledge and have more opportunities to put it into practice. With more successful experience, patients would experience more confidence in self-management. So the long disease duration played a positive effect on the relationship between health literacy and self-efficacy for managing chronic disease. Therefore, the third hypothesis was verified. Finally, the result indicated that disease duration plays a moderating role in self-efficacy for managing chronic disease and health outcomes. In this study, the impact of self-efficacy for managing the chronic disease on health outcomes in the long duration patients is more significant than those with short duration. The possible reason was that patients with long disease duration had more successful experience in self-management and maintaining calmness to cope with their diseases.

This study explored the relationship between self-efficacy for managing chronic disease and disease duration in health literacy and health outcomes. The study validated the mediation model, emphasizing the mediating effect of self-efficacy for managing chronic disease between health literacy and health outcomes among older patients with chronic disease. The mediation-moderated model proposed suggested that disease duration also affects the health outcomes of patients. The research enriches previous results and provides guidance for healthcare professionals. to improve older patients' health outcomes. Professionals could conduct self-efficacy-oriented inventions (e.g., peer support groups) interventions to enhance older adults' self-efficacy. Moreover, we recommend that professionals could consider the disease duration in patients when conducting interventions. We should strengthen the evaluation of health literacy and self-efficacy among the older with chronic diseases, especially those with short disease duration.

## Limitations

The findings in this study are subject to three limitations. First, it is a cross-sectional design that could not reveal the causes and effects of the relationship. Second, this study used a convenience sample from four hospitals in Zhejiang which limits generalizability. Third, the study just tested single mediating and moderating variables. Accordingly, longitudinal studies could be conducted in the future. In addition, future research could use a larger random sample. Furthermore, it will be important to explore the multiple moderating roles on health outcomes among older adults with chronic diseases, such as socioeconomic status.

## Conclusion

In conclusion, this study found that health literacy had a significantly positive association with health outcomes among older patients with chronic disease; self-efficacy for managing chronic disease mediated the relationship between health literacy and health outcomes; disease duration moderated the direct relationship between health literacy and health outcomes, as well as the second half of the mediating effect of self-efficacy for managing chronic disease.

## Data availability statement

The raw data supporting the conclusions of this article will be made available by the authors, without undue reservation.

## Author contributions

JL: data collection, data interpretation, and manuscript drafting. SS and YG: data collection and data interpretation. HL: data collection and data analysis. LF: data collection. XZ: study design and manuscript reviewing. HX: study design, conceptual interpretation, and manuscript reviewing. All authors contributed to the article and approved the submitted version.

## Figures and Tables

**Table A1:** The multiple group analysis.

	**CMIN**	**DF**	**CMIN/DF**	**NFI**	**CFI**	**GFI**	**RMSEA**
Unconstrained model	11.457	2	5.728	0.986	0.989	0.988	0.100
Measurement weight model	15.593	3	5.198	0.981	0.985	0.984	0.095
Structural weight model	27.217	6	4.536	0.968	0.974	0.973	0.087
Structural covariance model	33.810	7	4.830	0.960	0.968	0.966	0.09
Structural association error model	40.426	9	4.492	0.952	0.962	0.959	0.086
Measurement error model	43.376	11	3.943	0.948	0.961	0.956	0.079

**Table B1:** Model difference test.

	**Δ-CMIN**	**Δ-DF**	***P***	**Δ-NFI**	**Δ-CFI**	**Δ-GFI**
Measurement weight model	4.136	1	0.042	–0.005	–0.004	–0.004
Structural weight model	15.760	4	0.003	–0.018	–0.015	–0.015
Structural covariance model	22.353	5	<0.001	–0.026	–0.021	–0.022
Structural association error model	28.969	7	<0.001	–0.034	–0.027	–0.029
Measurement error model	31.919	9	<0.001	–0.038	–0.028	–0.032
